# Cost-effectiveness of the Da Qing diabetes prevention program: A modelling study

**DOI:** 10.1371/journal.pone.0242962

**Published:** 2020-12-31

**Authors:** Wanxia Hu, Wenhua Xu, Lei Si, Cuilian Wang, Qicheng Jiang, Lidan Wang, Henry Cutler

**Affiliations:** 1 School of Health Management, Anhui Medical University, Hefei, China; 2 Affiliated Stomatological Hospital, Anhui Medical University, Hefei, China; 3 The George Institute for Global Health, Sydney, New South Wales, Australia; 4 School of Public Health, Anhui Medical University, Hefei, China; 5 Centre for the Health Economic, Macquarie University, Sydney, New South Wales, Australia; Icahn School of Medicine at Mount Sinai, UNITED STATES

## Abstract

**Objective:**

The Da Qing Diabetes Prevention program (DQDP) was a randomized lifestyle modification intervention conducted in 1986 for the prevention and control of type 2 diabetes in individuals with impaired glucose tolerance. The current study estimated long-term cost-effectiveness of the program based on the health utilities from the Chinese population.

**Methods:**

A Markov Monte Carlo model was developed to estimate the impact of the intervention from the healthcare system perspective. The analysis was run over 30-year and lifetime periods and costs were estimated respectively as health management service costs. Baseline characteristics and intervention effects were assessed from the DQDP. Utilities and costs were generated from relevant literature. The outcome measures were program cost per quality-adjusted life-years (QALYs) gained and incremental cost-effectiveness ratio (ICER) of the intervention. Sensitivity analyses and threshold analyses were performed.

**Results:**

Using a 30-year horizon, the intervention strategy was cost-saving and was associated with better health outcomes (increase of 0.74 QALYs per intervention participant). Using a lifetime horizon, the intervention strategy was cost-saving and was associated with additional 1.44 QALYs. Sensitivity analyses showed that the overall ICER was most strongly influenced by the hazard ratio of cardiovascular disease event.

**Conclusions:**

The Da Qing lifestyle intervention in a Chinese population with impaired glucose tolerance is likely to translate into substantial economic value. It is cost-saving over a 30-year time and lifetime frame.

## Introduction

Diabetes is a prominent global public health issue and is expected to become one of the world’s most severe health problems within the next 25 years. According to 2017 data from the International Diabetes Federation, one in 11 adults worldwide has diabetes (425 million adults’ total), among whom half are undiagnosed [1]. The prevalence of diabetes has risen dramatically in recent years in developing countries, with particularly sharp increases in China [2]. The estimated overall prevalence of type 2 diabetes mellitus (DM2) in Chinese adults is 11.6% [3]. Furthermore, the prevalence of impaired glucose regulation (including impaired fasting glucose and impaired glucose tolerance, IGT) is estimated at 50.1% [3]. Economic losses due to diabetes and related cardiovascular diseases amounted to $557.7 billion in China from 2005 to 2015, and the medical and economic burden of diabetes thus constitutes a significant public health challenge for the country [4].

Evidences suggests that lifestyle modifying intervention for pre-diabetes can dramatically reduce the incidence of diabetes and its complications, both in developed and developing countries [5, 6]. Most mathematical models for non-pharmacological prevention of diabetes have been carried out in high-income areas rather than in developing countries with lower incomes and more limited health resources [7, 8]. The few studies conducted in China include economic evaluation of prevention, screening, and lifestyle modification interventions [9–11]. However, these studies did not consider appropriate health utilities based on the preferences of Chinese citizens [9].

The Da Qing Diabetes Prevention Program was a 6-year randomized trial of a lifestyle intervention for control and prevention of diabetes in individuals with IGT having an average age of 45 years (range 25–74) at enrollment, conducted in Da Qing, China from 1986 to 1992 [12]. The DQDP was the first randomise trial initiated in China to assess whether a lifestyle modification could delay onset of diabetes and serious complications in individuals with IGT. Over 30 years, DQDP was associated with a 26% reduction in cardiovascular events, and a 33% and 26% reduction in cardiovascular deaths and all-cause mortality, respectively [13]. Given the potential economic and public health benefits of investment in prevention and associated reductions in resource utilization, this study sought to assess the value of investment in the DQDP lifestyle intervention in the Chinese context for prevention of type 2 diabetes via cost-effectiveness analysis.

## Methodology

### Study design and population

We conducted a model-based economic evaluation of the DQDP trial from a health system perspective, comparing the lifestyle modification strategy with usual care in a population with IGT. The DQDP trial has been described in previous publications [12–15]. In brief, a total 576 participants were randomized at baseline to either the control group or one of the lifestyle intervention groups (diet, exercise, diet and exercise), and the three intervention groups were combined to one group (438 participants) for later follow-up studies. The intervention group was offered lifestyle support in addition to usual care. The intervention strategies consisted of a 6-year therapeutic lifestyle intervention and regular health screening services, which were provided by community healthcare workers in village or community healthcare institutions. The screening visit included screening for new symptoms, disease, blood glucose measurement. Lifestyle counselling was offered at a 3-month follow-up visit, as well as a physical examination at one year. The control group received usual pre-diabetes management without additional intervention.

### Diabetes disease model

**Fig 1:**
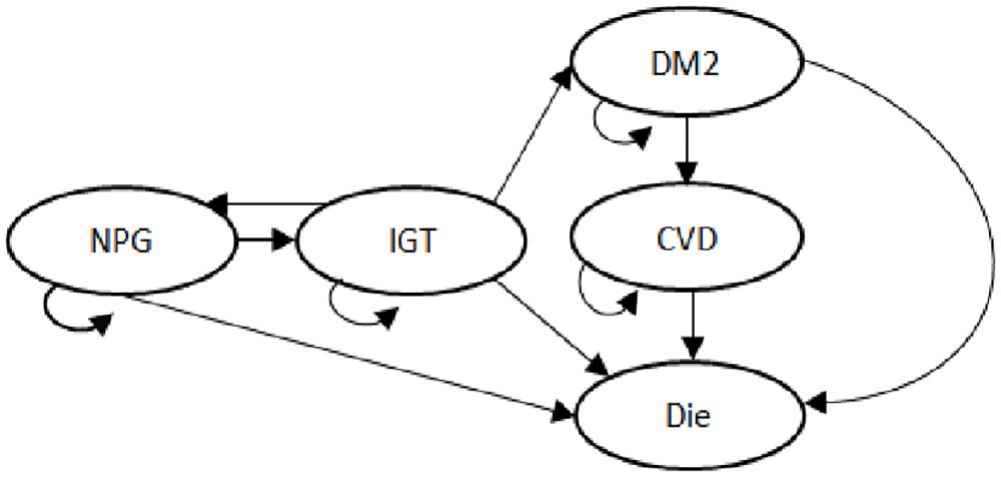
Structure of the diabetes state-transition model. Simulated patients transit in the model following the arrow direction. Simulation is concluded when all simulated patients transit to the ‘death’ state.

The study model has a Markov structure and includes annual transition probabilities between disease states. In addition to disease progression, the model tracks costs and QALYs. For our analysis, the model was developed to simulate long-term costs and clinical effectiveness of the interventions in a cohort of individuals with pre-diabetes under two main strategies: (1) DQDP active lifestyle intervention in addition to usual care, and (2) usual care. The one-year transition cycle moved from one health state to another among five health states: normal postprandial glucose (NPG), IGT, DM2, diabetes with cardiovascular disease (CVD), and death (Fig 1). A CVD event is defined as either myocardial infarction or stroke. Death included individuals who died from diabetes or any other cause. An individual could transition from their current health state to a different health state or remain in their current health state at the end of each one-year cycle in this Markov process.

Long-term modelling referred to a time horizon over a 30-year period and lifetime horizons beyond the six-year intervention. Compared to the usual care group, the DQDP intervention led to different transition probabilities between the five disease states, resulting in extra cost above those for usual care. The management of DM2 or CVD in these two groups was assumed to follow the same routine clinical treatment, and the costs and health effects of DM2 and CVD were the same for all subjects, regardless of which group they belonged to.

The selected model inputs and assumptions are described in Table 1. Clinical data required to inform the model structure and parameters were based on a combination of data from a 30-year outcome study and findings from published academic literature [13–15]. Non-CVD-specific mortality was calculated using 5-year age-specific and sex-specific Chinese life tables [16]. Diabetes-risk, CVD risk, diabetes-specific mortality, and CVD-specific mortality were derived from the 30-year follow-up study and converted into annual probabilities (p) using the following equation:

$$\text{P}=1-\text{exp}(\text{ln}(1-{\text{P}}_{\text{t}})/\text{t})$$

where the P_t_ is the cumulative risk over a period of years and t is the total number of years.

**Table 1:** Baseline values of parameters used in the model.

Parameters	Intervention group	Control group	HR (IN vs. C)	References
Epidemiological parameters				
Age at enrollment (years)	45.0 (9.3)	45.0 (9.3)		[12]
NPG to IGT	0.193 (0.175–0.275)	0.193 (0.175–0.275)		[17]
IGT to NPG	0.192 (0.185–0.200)	0.192 (0.185–0.200)		[17]
IGT to DM2				
45-65y		0.1410	0.57(0.41–0.81)	[13–15]
66y-		0.1113	0.61 (0.45–0.83)	[13–15]
Onset of CVD				
45-51y	0.009(0.005–0.013)	0.009(0.002–0.016)		[13–15]
76y-		0.0358	0.74 (0.59–0.92)	[13–15]
DM2 to death		RR (C vs. generation)		
		2.00(1.93–2.08)	0.74 (0.61–0.89)	[13–15, 18]
CVD to death		RR (C vs. generation)		
		2.13(2.01–2.26)	0.67 (0.48–0.94)	[13–15, 18]
All–cause mortality		Life table	0.74 (0.61–0.89)	[13, 16]
Unit costs (¥)				
Intervention cost	3374			[9]
Annual screening service	422			[19]
Diabetes treatment	6436	6436		[20]
CVD treatment	11680	11680		[20]
Utilities (age 45* )				
NPG	0.936	0.936		[21]
IGT	0.931	0.931		[22]
Onset of diabetes	0.925	0.925		[23–25]
CVD	0.779	0.779		[23–25]

Values are expressed as mean (standard deviation) or mean (95% confidence interval).

NPG, normal postprandial glucose; IGT, impaired glucose tolerance; DM2, type 2 diabetes mellitus; CVD, cardiovascular disease; HR, hazard ratio; IN vs. C, intervention versus control; RR, relative risk;

*Example shown for a person aged 45.

We validated the micro-simulation model, as has been done in previous studies [26]. Our study model was validated by comparing life expectancy generated by the mortality module and estimations from the Statistical Bureau of China for the same period (S1 Table). We carried out internal validation of the simulation model by testing its performance in replicating the incidence of CVD and mortality over 30 years of follow-up (Table 2).

**Table 2:** Health outcomes and results from cost-effectiveness analysis.

Strategy	Cost (¥)	LEs (years)	QALYs	Incr LEs (years)	Incr QALYs	ICER (C/QALY)
	(95% CI)	(95% CI)	(95% CI)	(95% CI)	(95% CI)	(95% CI)
30-year						
Usual care	79848 (50543,107539)	24.92 (16.64−28.77)	15.14 (10.70−18.01)			
Intervention	74510 (50948,95736)	26.36 (18.41−29.15)	15.88 (11.78−18.26)	1.44	0.74	−8211 (−24483,3366)
Lifetime						
Usual care	88241 (61578,151126)	27.60 (19.97−46.74)	15.85 (11.33−22.43)			
Intervention	86294 (63000,141427)	30.75 (21.73−49.15)	17.01 (12.35−23.12)	3.14	1.16	−1652 (−76257,1755)

CDV, cardiovascular disease; LE, life expectancy; CI, confidence interval; Incr, incremental; QALY, quality-adjusted life-year; ICER, incremental cost-effectiveness ratio.

### Costs

We accounted for direct medical costs associated with avoided DM2, including the costs of clinic visits, medications, and inpatient hospital admissions, as well as cost savings due to avoided DM complications. The health economic parameters were collected from published academic literature. Annual treatment costs of DM2 and CVD were estimated based on data from a cross-sectional study of 2054 adults in China [20]. The costs of the implementation and regular screening visit were also calculated as part of total intervention costs, and were assigned only to the intervention subjects. The initial implementation costs came from the published literature from the DQDP. The screening costs for NPG participants include the cost of initial 2 h post-glucose screening test for each 3-month visit. And costs of health management for IGT, DM2 and CDM patients include the screening costs for each 3-month visit, including the cost of the initial 2 h post-glucose screening test, confirmatory diagnosis from an oral glucose tolerance test in subjects who had a positive 2 h post-glucose test, and annual physical examination [12]. Additionally, we calculated the expenditures of community health workers implementing the health management for DM2 and CDM patients, given the consideration that providing these services would increase their workload. The unit labor cost was measured as actual expenditures based on the total costs and amounts of services [27]. All costs were accounted for and adjusted to the 2016 Consumer Price Index of China [28] (Table 1).

### Outcomes

The model used QALYs to quantify health outcomes. QALYs of individual subjects were calculated according to the time spent in different health states and the health utilities assigned to these states based on Chinese preferences (Table 1). For individuals with NPG, utilities value were determined as those of the general population at corresponding ages, and the time duration of utility was considered for the population according to the number of years with diabetes [21]. The utility value was adjusted as 99.08% of NPG [20]. The mean decrement in this study was -0.012 for DM2 alone and -0.158 for DM2 with CVD [23, 24]. Given variation in the impact of diabetes on individuals of different ages, age-specific health utilities were calculated with a decrement rate of 0.003 per year. For individuals with NPG aged 50 years, the calculation of utilities considered only the impact of age. In contrast, for subjects who had diabetes with CVD, the calculation of utilities included coefficients for age, impact of DM2, number of years’ duration of DM2, and complications (Table 1). For example, the health utility of a subject with DM2 and CVD for 10 years at the age of 60 was calculated as 0.644, as derived from the utility of diabetes (0.832), the decrements of stroke (−0.158) and subtracting 0.03 [10 × (−0.003)].

### Statistical and sensitivity analysis

Cost-effectiveness analysis was performed using base-case and sensitivity analyses. In the base-case analyses, we estimated the costs and incremental costs per QALY gained over 30-year and lifetime horizons after the intervention. Furthermore, ICERs were calculated in terms of costs per QALY gained for intervention subjects compared to usual care in order to capture health improvement and cost differences. The population in each health state in the model was assigned a QALY weight and annual healthcare costs. Costs and QALYs were discounted at a rate of 3% [29]. Half-cycle correction for both costs and health effects was applied to the model [29]. Cost-effectiveness analysis was performed with Tree Age Pro Healthcare 2019 (Tree Age Software, Inc., Williamstown, MA, U.S.).

One-way and probabilistic sensitivity analyses were performed for the parameter uncertainties. All key variations in the study may influence the performance of the intervention, costs, utilities, and discount rates were analyzed. In the one-way sensitivity analysis, we varied the cost parameters and utility weights associated with IFG by ±20%, and we varied the discount rates from 1–5%. Risk reduction of the intervention on diabetes and CVD was estimated, including distributions and 95% confidence intervals [30]. Probabilistic sensitivity analyses were conducted using Monte Carlo simulations of 1000 iterations. The initial intervention costs were fixed values, as data were calculated from pertinent literature. To interpret cost-effectiveness results, the ICER was compared to a willingness-to-pay (WTP) threshold for China, set at ¥37446 per QALY gained (63% of the annual per-capita GDP for China in 2016) [31, 32].

## Results

The cumulative incidence of DM2 and all-cause mortality over a 30-year period were 97.1% and 56.3% respectively in controls, whereas the rate of diabetes onset and all-cause mortality were 49% and 26% lower respectively in the intervention group (cumulative all-cause mortality of 45.7%). The mean delays to onset in the intervention group compared to the control group were 4.04 and 1.77 years for DM2 and CVD respectively (Table 2), as the reported in the 30-year follow-up study [13]. Thus, the model fit was good and simulation outcomes were reliable.

### Outcomes and cost-effectiveness for participants

Health outcomes in the intervention and control groups are described in Table 2. Over a 30-year period, the delayed onset of DM2 led to improvements of 1.44 non-discounted life-years and 0.74 QALYs in the intervention versus the usual care group. Over a lifetime horizon, the intervention was associated with an increase in average overall survival of 3.14 years and 1.16 QALYs.

**Fig 2:**
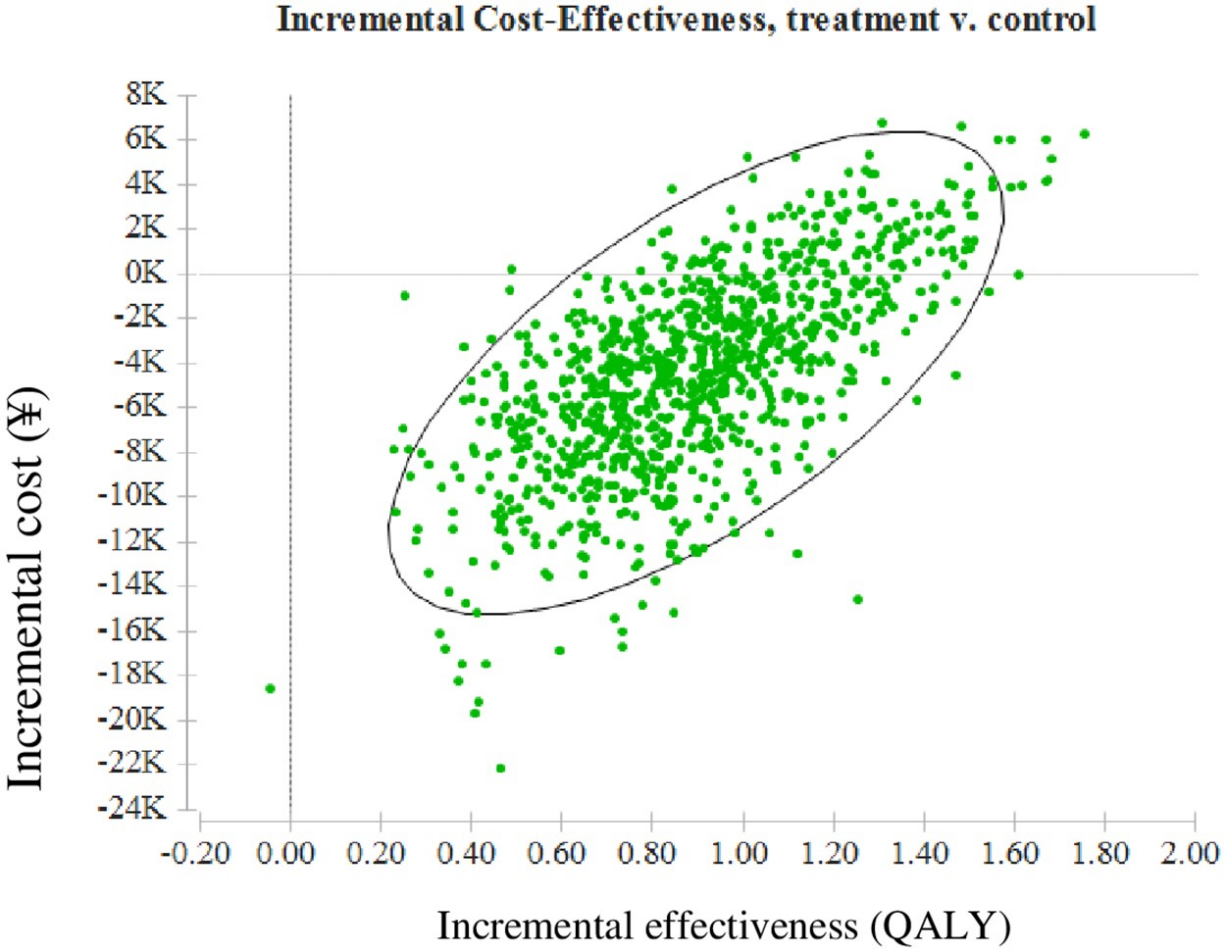
Lifetime incremental cost-effectiveness scatters plot. QALY, quality-adjusted life-year.

As shown in Table 2, the total savings of the intervention were ¥5338 per participant over 30 years and ¥1921 per person over their lifetime. The ICER of the intervention group was ¥7238 and ¥1652for intervention participants over a 30-year and lifetime horizon, respectively, compared to individuals receiving usual care. The lifetime incremental cost-effectiveness scatters plot was shown in Fig 2.

### Sensitivity analysis

**Fig 3:**
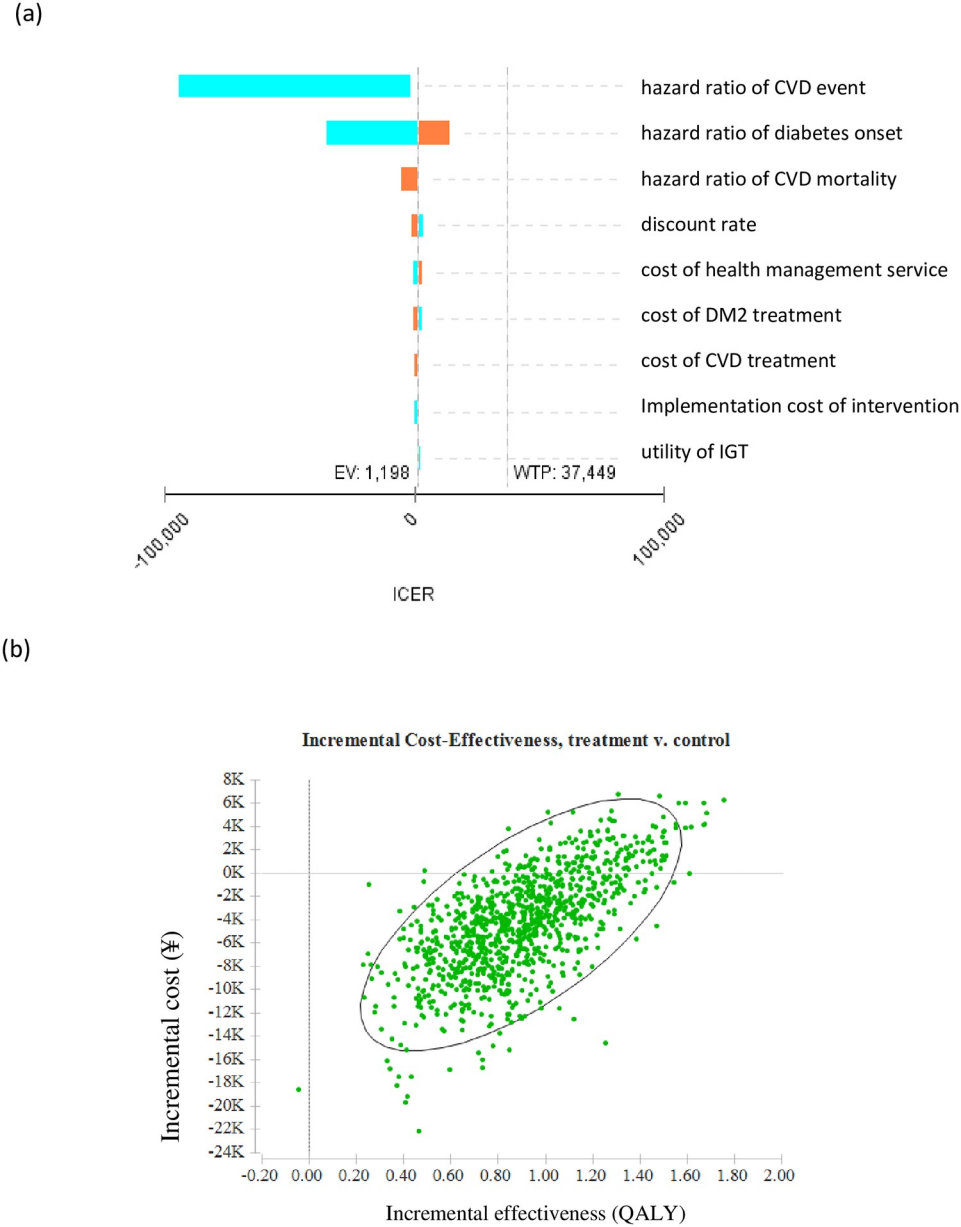
(a) Tornado diagram showing changes in incremental cost-effectiveness ratio attributable to various predictors. EV, expected value of ICER, and was automatically; WTP, willingness-to-pay. (b) Lifetime incremental cost-effectiveness scatters plot. QALY, quality-adjusted life-year.

The results of deterministic sensitivity analyses over lifetime are presented in Fig 3 and Table 3. The tornado diagram in Fig 3 shows the percentage change in ICER for key variables. Key factors impacting ICER calculations in one-way sensitivity analyses included the hazard ratio of a CVD event, discount rate, annual treatment costs of diabetes, the utility of IGT, and the incremental screening costs (Table 3). The intervention strategy was cost-saving when these key parameters varied within the range tested. The discount rate had the largest impact on total health effectiveness, followed by utility of IGT, and hazard ratio of CVD event. The ICER was ¥2788 when the discount rate was varied to 5%. The discount rate also made the largest contribution to the total costs per patient, followed by treatment costs of diabetes and CVD, and hazard ratio of DM2 onset. And the hazard ratio of CVD event had the largest impact on ICER, followed by hazard ratio of DM2 onset and the hazard ratio of CVD mortality. When the hazard ratio of DM2 onset of the intervention group was 0.83 times that of the control group, the ICER of the intervention strategy was raised to ¥11218 per QALY gained, which is still lower than the WTP threshold. The intervention strategy was more cost-effective when expenses related to DM2 and CVD were higher. For same intervention carried out in a population with normal postprandial glucose, the incremental QALY and ICER of the intervention strategy are estimated at 1.09 QALYs and ¥429 per QALY gained, respectively.

**Table 3:** One-way sensitivity analysis over lifetime period.

		Incr costs	Incr effectiveness	ICER(¥/QALY)
		(¥)	(QALY)	
Base case		-1912	1.16	`-1652
Implementation costs	+20% (4049)	-1246	1.16	-2602(-22905,4067)
	−20% (2699)	-3026	1.16	-1785(27701,2729)
Health management costs	+20% (506)	-519	1.16	-446(-21339,3692)
	−20% (338)	-3323	1.16	-2857(26524,1900)
Diabetes treatment costs	+20% (7723)	-3078	1.16	-2647(-32787,1723)
	−20% (5149)	-96	1.16	-82(-16098,3603)
CVD treatment costs	+20% (14106)	-2636	1.16	-2267(-25926,3206)
	−20% (9344)	-557	1.16	-496(-18092,3574)
Discount rate	1%	-485	1.97	-246(-23149,4656)
	5%	2001	0.72	2788(-29302,3172)
Utility weights associated with IGT	+20%	-3992	1.05	-3805(-17873,4670)
	−20%	1921	0.78	-2477(-121533,6827)
Effectiveness of intervention				
Hazard ratio of DM2 onset	0.45	-13084	1.35	-9669(-59688,-5444)
	0.83	10858	0.97	11218(7976,19620)
Hazard ratio of CVD event	0.59	-4094	1.29	-3171(-138159,-2220)
	0.92	-1337	1.15	-1162(-21198,1887)
Hazard ratio of CVD death	0.48	3210	1.43	2243(-11199,5344)
	0.94	-3073	1.10	-2792(-30266,2788)
Scenario analysis				
Universal health screening of individual with NPG		470	1.09	429(-18524,4318)

IGT, impaired glucose tolerance; Incr, incremental; CDV, cardiovascular disease; QALY, quality-adjusted life-year; NPG, normal postprandial glucose.

## Discussion

To our knowledge, this is the first economic evaluation of a large community-based clinical trial of a lifestyle intervention strategy for diabetes prevention in China. In addition to the main 30-year follow-up data, all other data included into the study model were obtained from Chinese data sources. Our analyses are thus preferable to the other models based on health utilities and transition probabilities from more developed countries. By including information on costs related to health management, our study will enable policymakers to make better-informed decisions for future diabetes prevention.

Earlier detection and treatment of diabetes is associated with delay and prevention of complications [33, 34]. Our study found a lifetime improvement of 1.16 QALYs for individuals who received the intervention at age 45, with a 30-year improvement of 0.74 QALYs. In comparison to the 30-year time horizon, there was no notable extra improvement in life expectancy. This may be due to a rapid increase in mortality among people aged 75 years and older, as participants were aged 45 years on average at the time of the intervention, and the overall life expectancy in China in 2016 was 76.5 years [35]. Furthermore, as reported in follow-up studies from the DQDP, the widening difference in cardiovascular mortality between intervention and control participants became statistically significant only 23 years following the intervention [15]. Thus, our findings may be explained in part by the use of 30-year follow-up data to simulate lifetime outcomes.

Findings from the current study regarding improvements in quality-adjusted life-years are consistent with those of a systematic review, finding that lifestyle interventions were associated with a median increase of 0.159 QALYs (range: 0.003–2.91) in individuals with a high risk of diabetes [8]. Our results show the DQDP to be better than similar diabetes prevention programs in Sweden (increase of 0.43 QALYs for males and 0.45 QALYs for females among individuals aged 50 years with pre-diabetes over lifetime) [5] and the U.S (increased 0.14 QALYs for lifestyle) [36]. These differing findings may stem from the simulation of effects of the intervention over only 10 years in these two studies, as well as from higher life expectancy and higher utility weights in the Sweden and U.S. compared to China. As reported in previous study, health utility differences between countries remain substantial [37]. Health utilities used in previous cost-effectiveness studies on lifestyle interventions for diabetes prevention were based on preferences from industrialized countries, whereas the utility values in our study were derived from Chinese data. Compared to developed countries, China tends to assign higher utilities to the same health state and more QALYs for the same life expectancy. Such differences in utility ratings can have substantial impacts on estimation of QALYs [25]. Accordingly, the use of utility values reflecting the health preferences of local populations in cost-effectiveness analyses is important.

The lifetime estimated ICER for health management costs in this study (-¥1652 or US$249 per QALY gained) is economically attractive by health economic evaluation standards [29], and is substantially better than cost-effectiveness estimates for many diabetes prevention programs worldwide. The median ICER observed from a health system perspective over a period of ≥ 25 years post intervention was £2976/QALY [8], and our results even show cost savings similar to those observed in Australia (with cost saving of $289) [38]. The cost savings of the DQDP compared to other diabetes prevention programs can likely be explained by the use of a 6-year community intervention in a developing country, including group counseling and intervention rather than more costly one-on-one services. Benefits stem from the long-term effects of delayed or averted diabetes and related complications, through which a 6-year intervention can bring about substantial medical cost savings in China.

The ICER was most strongly affected by the hazard ratio of diabetes onset, possibly on account of the wide range of this variable and its further effects on onset and mortality from diabetes complications. Delayed diabetes onset thus led to reduced medical costs from diabetes and its complications. Our findings from the threshold analysis appear promising and confirm the capacity of the DQDP to modify patients risk of diabetes and bring about population-wide reductions in medical complications and future costs.

The DQDP prevention program was cost-saving over 30-year time and lifetime period. The national basic public health service program is a major component of China’s 2009 primary healthcare system reform, which implemented basic public health services with the aims of equity and access [27]. Under this reform, primary healthcare institutions are responsible for providing basic health services, including but not limited to health management services for major chronic diseases such as hypertension and DM2. In the present study, we did not address the possibility of confounding from this policy, and we calculated the intervention costs based on labor expenditures and health management service costs after the reform. Under China healthcare reform, health management services will be universally available and will have enriched scope and content [27]. Accordingly, extrapolated estimates of health management costs will offer a more accurate representation of true costs and will thus provide more valuable information for decision-makers.

Given limited budgets available for diabetes prevention, it is imperative that policymakers have the best information available in order that healthcare funds be most effectively allocated. Given the high absolute burden of disease related to diabetes, lifestyle-based diabetes prevention efforts can lead to meaningful improvements in health and are likely to be worthwhile investments [2, 39]. Furthermore, prevalence and incidence rates of diabetes as well as pre-diabetes have increased dramatically in recent decades, especially among younger people [40, 41]. Keeping in mind that diabetes prevention efforts are generally initiated in industrialized countries at age 45 or 55 years [7], we recommend that the Chinese government expand funding for management and prevention of diabetes, especially for younger people and other high-risk populations, and that the role of chronic disease prevention and management be strengthened in health-related policies and guidelines.

### Limitations

Several limitations to our study must be acknowledged. First, the economic burden assessed did not include non-medical direct or indirect costs such as loss of income and time for individuals with diabetes and their families. Accordingly, our analyses may in fact underestimate the economic effects of the intervention. Second, we did not include underlying assumptions concerning microvascular outcomes such as changes associated with retinopathy, neuropathy and nephropathy. We recommend that future analyses be conducted using this data set and incorporating these microvascular outcomes, which will permit better comparability between studies. Thirdly, we estimated the long term cost-effectiveness for diabetes prevention based on health outcome data from follow-up studies of the DQDP, and did not address the effects of other health policies over the study time period.

## Conclusion

Through a robust cost-effectiveness analysis, our study showed that the Da Qing Diabetes Prevention Program for individuals with IGT had a positive impact in terms of increased QALYs, that cost savings over a 30-year time period were substantial, and that the program has high lifetime cost-effectiveness in China. These findings have implications for prioritizing and targeting similar interventions aimed at prevention of chronic diseases.

## Supporting information

S1 Table Life expectancy generated by statistical bureau of China and the model. (DOC)

S1 Appendix CHEERS checklist. (DOC)

## Abbreviations

CI: confidence interval
CVD: cardiovascular disease
DM2: type 2 diabetes mellitus
DQDP: Da Qing Diabetes Prevention Program
ICER: Incremental cost-effectiveness ratio
IGT: impaired glucose tolerance
LE: life expectancy
NPG: normal postprandial glucose
QALY: quality-adjusted life-year
WTP: willingness-to-pay
