# Supplementary material for: Cost-effectiveness of the Da Qing diabetes prevention program: A modelling study
Source: PLoS One. 2020 Dec 31;15(12):e0242962. doi: 10.1371/journal.pone.0242962 (PMC7774969; doi:10.1371/journal.pone.0242962)
Supplement: S1 Table — (DOC) [file pone.0242962.s001.doc]

**S1 Table. Life expectancy generated by statistical bureau of China and the model**

| Age (year) | Mortality | Probabilities of live | Number of survival | Person-year of survival | Life expectancy (year) by SBC | Life expectancy (year) by the model |
| --- | --- | --- | --- | --- | --- | --- |
| 0 | 0.004694 | 0.995306 | 100000 | 99766 | 74.69 | 74.80 |
| 40 | 0.002155 | 0.997845 | 96149 | 96045 | 37.81 | 37.62 |
| 50 | 0.004680 | 0.995320 | 93333 | 93115 | 28.81 | 28.89 |
| 60 | 0.010322 | 0.989678 | 87562 | 87113 | 20.34 | 20.39 |
| 70 | 0.030434 | 0.969566 | 74274 | 73161 | 13.00 | 13.06 |
| 80 | 0.084522 | 0.915478 | 46412 | 44530 | 7.51 | 7.63 |
| 80 | 0.084522 | 0.915478 | 46412 | 44530 | 7.51 | 7.63 |

SBC: statistical bureau of China
