# Supplementary material for: Cost-effectiveness of the Da Qing diabetes prevention program: A modelling study
Source: PLoS One. 2020 Dec 31;15(12):e0242962. doi: 10.1371/journal.pone.0242962 (PMC7774969; doi:10.1371/journal.pone.0242962)
Supplement: S1 Appendix — (DOC) [file pone.0242962.s002.doc]

**S1 Appendix**

**CHEERS Checklist—Items to include when reporting economic evaluations of health interventions**

| **Item** | **Reported on page No** |
| --- | --- |
| **Title and abstract** |  |
| Title  Abstract | 1-2 |
| **Introductions** |  |
| Background and objectives | 3-4 |
| **Methods** |  |
| Target population and subgroups | 4 |
| Setting and location | 4 |
| Study perspective | 3 |
| Comparators | 4-5 |
| Time horizon | 4 |
| Discount rate | 7 |
| Choice of health outcomes | 6-7 |
| Measurement of effectiveness | 8 |
| Measurement and valuation of preference based on outcomes | 6-7 |
| Estimations resources and costs | 5-6 |
| Currency, price data and conversion | 6 |
| Choice of model | 4-5 |
| Assumptions | 4, Fig. 1 |
| Analytical methods | 7 |
| **Results** |  |
| Study parameters | 8,Table1 |
| Incremental costs and outcomes | 8-9, Table 2 |
| Charactering uncertainty | 9, Fig. 2,Table 3 |
| Characterizing heterogeneity | 9-10  Figs. 2 and 3 |
| **Discussion** |  |
| Study findings, limitations, and current knowledge | 10-14 |
| **Others** |  |
| Sources of funding | 14 |
| Conflicts of interest | 15 |
